# Expulsion of Two Fœtuses of Unequal Size at the Same Time, with Remarks on Superfœtation

**Published:** 1848-02

**Authors:** Elias Horlbeck


### RECORD OF MEDICAL SCIENCE.

*Expulsion of Two Fœtuses of unequal size at the same time, with remarks on Superfœtation.* By ELIAS HORLBECK, M. D.—On 27th June, 1847, I was requested to visit Maria, ill with an alarming flooding which had been brought on from undue exertion. Found the patient in an exhausted condition, the floor being washed with blood. They stated to me that she had passed two menstrual periods, and was supposed to be about two months advanced in pregnancy. Appropriate means were resorted to, among which was the use of the tampon, which succeeded in arresting the hemorrhage and the woman made a rapid recovery; as it was not in my power to examine the coagula, I concluded that she had lost the product of conception, never having seen a case in which so much blood had been lost where it had been preserved. This, it appears in the sequel, was not the fact, and she went on suffering from occasional small hemorrhages until I was again called to see her, October 16th, three months and nineteen days after the first attack, with profuse flooding, which had commenced two days previously. She stated that she had increased in size and in regular course, had felt the movement of the child, that her breasts and abdomen had enlarged with all the other symptoms of gestation. The os uteri was open, pains were occurring at regular intervals, and things had gone so far that I administered ergot to hasten the discharge of the embryo; after the first dose a well grown fœtus of between five and six months was expelled, which her mistress informed me palpitated some time after its birth. The flooding was now checked, and the placenta was in the course of the evening discharged. On dissection it exhibited the following appearances: the placental mass was of considerable size, having a large ruptured sac on one side, lined with the amnion, from the centre of which sprung the umbilical cord. The maternal surface of the whole mass, presented a fresh appearance, as if just separated from the uterus; on close examination, it could be divided into two unequal masses, connected with the smaller of which was perceived a smaller fœtus enveloped with its usual coverings. The transparent amniotic covering contained a clear fluid, in which was floating a fœtus some seven or eight lines in length. The sac being opened, the embryo was recognized, sound, fresh and plump and life-like in its whole appearance, presumed from its development to be about six weeks old. The features of the face, such as the eyes, mouth and nose, were distinctly visible, its thoracic and abdominal members about two lines in length, its abdomen completely closed. The umbilical vesicle was distinctly visible and filled with fluid. The cord had no swellings or bulgings in its course, but was of the same size throughout.

The circumstance of two fœtuses, of such disproportionate dimensions, having been expelled from the uterus at the same time, is of rather rare occurrence, and presents some interest as connected with the Theory of Superfœtation. How are we to account for their different degrees of development? Why has one reached the growth usual at between five and six months, and the other, only that of six weeks? Two theories present themselves in explanation of the phenomenon; the one, that twins were originally conceived, and that one of them in consequence of the flooding that occurred six or eight weeks afterwards, was blighted or arrested in its growth, and remained unchanged until both were discharged. The other, that it arose from superfœtation ensuing when the woman was about four months advanced in pregnancy. The facts of the case are curious on either view, and arguments may be urged in favour of one or the other. For the purpose of bringing the subject before you, I shall assume the truth of the latter hypothesis, and sum up the points in its favour. It is difficult then to conceive how so soft and perishable an object as the smaller fœtus could have been preserved in the perfect condition in which it was found, if we allow it to have perished at the first flooding, i. e. three months and nineteen days before it was passed away, for I presume it will hardly be contended, that it still continued to preserve its vitality and to remain undeveloped. I am aware that it not unfrequently happens that one fœtus becomes blighted and deprived of life and remains a long time (the access of atmospheric air being prevented) without undergoing decomposition. But in all these cases it presents a wrinkled or shrivelled appearance, as if it had been sobbed in water, or it has some evident marks of atrophy about it. Besides, if the fœtus perished at that time, the placenta connected with it would have manifested some morbid condition; on the contrary, it was covered with blood, as if it had been freshly separated, and preserved every manifestation of health with no decay, no hydatids. There is no doubt that we occasionally meet with instances of imperfectly developed embryos, discharged either before a full grown child, or retained until the full period of utero-gestation, and born at the same time with it, or within a few hours of it. These have been improperly attributed to superfœtation, but evidently result from disease or death of one ovum in a twin pregnancy, and are explicable in that way, without seeking any farther for the cause. It is admitted by most physiologists of the present day, that superfœtation in the human subject may take place under the following circumstances. They allow that one or more ovules may be fecundated within the period intervening between a first conception and the formation of the decidua. M. Orfila extends the time even up to the period when the ovule reaches the uterus, forgetting, as Velpeau remarks, that the anhistous membrane already presents a physical obstacle to the introduction of the seminal fluid. If, however, the opinion advanced by Sharpey, in the examination of a woman, who died in the third week of pregnancy, and since confirmed by Bischoff, Coste and others, be true, there is no such improbability in this opinion, viz: that the membrane decidua in the female, as well as in the bitch, is merely the ordinary mucous membrane considerably developed, and that it consists of enlarged uterine follicles and their blood-vessels, together with an unusual large secretion which these follicles have thrown out, and, according to Deschamps, this membrane is continuous with the lining of the fallopian tubes and the vagina; if, we say, this be true, there is no such improbability in M. Orfila's opinion. This interesting matter, opposing the views of Hunter, which have been so long time received by the profession, I perceive by the last Journals has been adopted, and confirmed by Coste, whose name is sufficient evidence of the attention he has given the subject. This form of superfœtation may be often the case in the regular course of events, but it can only be verified when two castes of the race are concerned, the double offspring being born at the same time and having equal developments.

It is also allowed that superfœtation may take place where there exists a double uterus, having separate openings into the vagina. In M. Cassari's Recherches sur les cas d'uterus double et de Superfœtation, forty-one instances of this formation have been collected from different sources, and particularly a remarkable one occurring to Madame Boivin, leaving no doubt of a subsequent conception having occurred. Since this publication, eight or ten others have been added to the list. In Muller's Archives for 1836, there is a case related in which a four months child was born six weeks after marriage, and mature twins forty weeks after the same event.

With the instances of uterine conception ensuing on extra uterine fœtation, I have nothing to do on this occasion, except that numerous cases of its occurrence confirm the fact, that ova are discharged during the period of gestation; a circumstance essential to the very existence of our theory. Quite a remarkable event of this kind is detailed in the Nouveau Journal de Medecine, where, after sudden death, a five months extra uterine, and a three months uterine fœtus were discovered to co-exist.

But the class of cases most difficult to be explained, and which offer the strongest confirmation of superfœtation in a single uterus, are those in which the birth of one mature child has been succeeded after the lapse of some weeks or months, by the birth of another. Several attested observations of this kind can be cited. Of the number, is one related by Dewees, as occurring to himself, in his favourable consideration of this subject, in the Medical Museum. The opponents of the doctrine, suppose that in such persons, both fœtuses were begotten at the same time, and that the tardy birth of the latter was owing to its slower development. But this explanation, as Churchill remarks, requires previous proof; that a slow growth of the fœtus involves a protracted gestation. The truth is, no theory as yet advanced is adequate to explain such a phenomenon in the normal condition; superfœtation being opposed by material obstacles, insurmountable in the present state of the science. In fact, whether fecundation of the ovule is effected by the action of spermatozoa or by the imbibition of the liquor seminis; whether the situation of its occurrence be in the ovary, as is generally believed; or, according to recent researches, the ovula periodically eliminated from the ovaries, descend to meet the fecundating fluid; or, according to Carpenter, the point of conjunction varies, it seems necessary that the semen should penetrate at least as far as the uterine cavity, in order that conception should take place. Now, soon after a successful copulation, the internal surface of the uterus becomes lined with the decidua, and the internal between the os internum and os tincæ is plugged up with a secretion from the glands, presenting a mechanical obstruction to the penetration of any thing from without, and opposing a barrier to the contact of the male and female principles. All this, I readily admit to be the situation of affairs, in a perfectly healthy progression of events, and in such there is a general expression of opinion against surperfœtation. But there may occur deviations from the regular order, sufficient to remove some of these difficulties. Let us examine the history of our case, to see if they did not exist there.

The flooding which she first experienced from its extent, must have proceeded from a separation of a portion of the placenta at the fundus uteri; and the blood in escaping, dislodged the plug of the os uteri, not however to such an extent as to prevent the progress of gestation; for we find that after the arrest of the flooding, she advanced towards the term with occasional losses, sufficient to keep up a separation of the parts; and it is not improbable that at one portion the membranes were never re-united to the inner surface of the uterus, or were connected by the feeble medium of coagula only. Here then we have an open os-uteri, and a passage by which contact might take place. When we now reflect on the history of the spermatozoa,—the tenacity with which they adhere to mucous surfaces; their vitality and activity; their enterprise in making their way through much greater obstacles, as in a partially closed vagina, or through fistulus openings in completely closed vulva; we may safely infer that no physical impediment existed in the present case to their penetrating into the cavity of the uterus, or so far as it was necessary to effect their object. The only difficulty now to be overcome, is the fecundation of an ovum and its after descent into the uterus.

The laws by which the perfecting of ova are completed, and the period of their ripening, is not yet understood. In general, it is required that one ovum should be displaced from the uterine organs before another can be matured, but this rule, like all others, has its exceptions, and it is only sufficient for me to recal the fact that subsequent conception does occur in cases where the double uterus formation exists, as also during the presence of extra-uterine fœtation. These conditions prove the possibility of an ovum being discharged, even though a female be enciente. This occurring, I know of no obstacle to prevent its after passage to the uterus. In the case recorded by Dr. Robert Lee, of death in the early period of pregnancy, the fallopian tube is reported as being open and unobstructed. Once arrived at their entrance, the stimulus of distention will cause it to encroach upon the cavity, which, with its growth, will soon give it a position in which to develope itself by the side of the other; and I see no objection even that in its enlargement, it should be connected with the other placenta, as was the situation of things in the case described, and which is so often remarked in ordinary twin cases.

The anatomical objection has been made against superfœtation, that as the gravid uterus enlarges, the fallopian tubes lie parallel to its sides, instead of running in a transverse direction towards the ovaria, as in the unimpregnated state, and that if an ovum be generated, the tubes could not use it, and it would fall into the cavity of the abdomen, or remain in the ovary. This objection may hold good in the very advanced periods, but does not exist until after the fourth month, and is therefore more specious than true, since in the double uterus cases I have alluded to, the reverse was the fact. Whether you allow the truth or falsity of my hypothesis, I hope you will find the details of the case, not entirely devoid of interest. I regard it as one of those occurrences, which, in the present want of positive information in relation to the phenomena of conception and generation, throw a shade of probability on the occasional possible occurrence of superfœtation.—*Charleston Med. Jour.*

